# Siwu Granules and Erythropoietin Synergistically Ameliorated Anemia in Adenine-Induced Chronic Renal Failure Rats

**DOI:** 10.1155/2019/5832105

**Published:** 2019-12-14

**Authors:** Yansheng Wu, Qiang Wan, Liqiang Shi, Jiaoying Ou, YingQiao Li, Fei He, Huiling Wang, Jiandong Gao

**Affiliations:** ^1^Department of Nephrology, Shuguang Hospital Affiliated to Shanghai University of Traditional Chinese Medicine, TCM Institute of Kidney Disease, Shanghai University of Traditional Chinese Medicine, Shanghai Key Laboratory of Traditional Chinese Clinical Medicine (14DZ2273200), Key Laboratory of Liver and Kidney Diseases (Shanghai University of Traditional Chinese Medicine), Ministry of Education, No. 528 Road ZhangHeng, Shanghai 201203, China; ^2^Department of Internal Medicine, Shanghai TCM-Integrated Hospital, Affiliated to Shanghai University of Traditional Chinese Medicine, No. 184 Road BaoDing, Shanghai 200082, China; ^3^Department of Nephrology, Traditional Chinese Medicine Hospital of Langfang City, No. 108 Road North Yinhe, Langfang 065000, China; ^4^Department of Nephrology, Xiamen Hospital of Traditional Chinese Medicine, No. 1739 Road Xianyue, Xiamen 361009, China

## Abstract

**Objective:**

Renal anemia in patients with end-stage chronic kidney disease is closely related to the deterioration of cardiac function, renal function, and quality of life. This study involved adenine-induced renal anemic rat models and evaluated the treatment effect of Siwu granules and/or erythropoietin (EPO).

**Methods:**

Fifty SD rats were randomly divided into 5 groups: control, model, Siwu, EPO, and Siwu plus EPO groups. The expression levels of NO, MDA, SOD, CAT, IL-6, TNF-*α*, EPO, EPOR, *α*-SMA, and TGF-*β*1 were detected in rats after 8 weeks of treatment with Siwu granules and/or EPO.

**Results:**

After modeling, 47 rats entered the stage of treatment. Siwu plus EPO treatment significantly increased the rat hemoglobin content (*p* < 0.05) and reduced blood urea nitrogen (*p* < 0.05) and serum creatinine (*p* < 0.001). Compared with the control group, the expression of EPO and EPOR in the kidney of rats with renal failure was significantly decreased (*p* < 0.05). Moreover, the Siwu plus EPO group improved the level of oxidative stress in rats with chronic renal failure and reduced the expression of inflammatory factors. The expression of *α*-SMA and TGF-*β*1 in rats with renal failure was higher, but there was no expression in the control group.

**Conclusion:**

Combined treatment of Siwu granules with EPO increased the expression of EPO and EPOR in the renal tissues and inhibited oxidative stress and inflammatory factors, improving the renal function and anemia.

## 1. Introduction

Anemia is an early sign and a common and severe complication of chronic kidney disease (CKD), especially in the end-stage renal disease (ESRD). In general, renal anemia occurs mainly due to the absolute deficiency of erythropoietin. Anemia in CKD is typically normocytic, normochromic, and hypoproliferative. Anemia of CKD is a multifactorial process due to relative EPO deficiency, uremic-induced inhibitors of erythropoiesis, shortened erythrocyte survival, and disordered iron homeostasis [1]. Renal anemia can lead to glomerular and tubular interstitial fibrosis [2]. This in turn affects the heart function [3], resulting in cardiac remodeling [4]. In addition, the subjective symptoms of anemia can aggravate the patient's discomfort, fatigue, and malaise and consecutively reduce the quality of life of patients. Oxidative stress is an important factor that causes inflammation in renal anemia patients. Oxidative stress, especially in the maintenance of hemodialysis patients, is related to the inflammatory state closely. Interaction between these conditions in patients leads to decreased EPO sensitivity. Oxidative stress weakens the redox reaction and shortens the life of red blood cells (RBCs), resulting in the increased production of hepcidin, reduced intestinal absorption of iron, blocked mobilization of iron storage, and decreased induction of iron transporter expression and erythropoietin resistance [5]. Subcutaneous injection of recombinant human erythropoietin (rhEPO) is the main means of treatment for renal anemia and has been in clinical use for many years. But due to its expensive treatment schedule, the use of rhEPO is limited [6]. Long-term injection of erythropoietin increased the blood viscosity and increased the risk of thrombosis, causing hyperkalemia, hypertension, and other side effects [7]. Therefore, to obtain ideal curative effects, and reduce the side effects with long-term injection of EPO, it is necessary to seek a suitable adjuvant. Recently, expert opinion also recommended that while rhEPO is presently an important mainstay erythropoiesis-stimulating agent, acting as EPOR (erythropoietin receptor) agonists, and/or selectly act through downstream EPOR pathways to bolster erythroid cell formation. Such agents could lessen rhEPO dosing, side effects, and/or costs [8]. Siwu decoction is a traditional Chinese medicine (TCM) and classical prescription for blood circulation. Modern research showed that Siwu decoction has clinical role in endocrine regulation, anemia correction, immune regulation, antifree radical, anti-inflammation, antidrug damage, and antiradiation injury, which improves the blood rheology and mutation functions [9]. More and more evidence, both in vivo and in vitro, supported the effect of Siwu decoction on anemia. Siwu decoction significantly increased the number of red blood cells, hemoglobin concentration, and EPO expression in 5-fluorouracil-induced anemic mice [10]. Siwu decoction also increases the differentiation and proliferation of bone marrow hematopoietic stem cells and thymocytes [11, 12].

Also, Siwu decoction is effective for the treatment of chronic renal failure [13]. To clarify the effect and mechanism of Siwu decoction in the treatment of renal anemia, this study used Siwu granules, including *Angelica*, *Ligusticum*, and *Rehmannia glutinosa*, which are sold in the market and complied with the quality control standards instead of decoction to minimize the influence of subjective and objective factors. We used rhEPO combined with Siwu granules to treat anemia in adenine-induced chronic renal failure rats to achieve the best therapeutic effect of kidney protection. Through protecting kidney injury, anemia caused by renal failure can be directly treated. Therefore, this study focuses on the role of Siwu and EPO in protecting the kidneys, which can ameliorate renal anemia.

## 2. Materials and Methods

### 2.1. Experimental Reagents and Drugs

The following experimental reagents and drugs were obtained: adenine (Shanghai Ronghe Pharmaceutical Technology Development Co., Ltd., 151029), anti-EPO antibody (Abcam, ab61224), anti-EPOR antibody (Abcam, ab61162), anti-TGF-*β*1 polyclonal antibody (AbSci, #AB41494), anti-*α*-SMA antibody (Boster, BM0002), Siwu granules (Jitai'an (Sichuan) Pharmaceutical Co., Ltd., Chinese medicine prescription: Z20020016), recombinant human erythropoietin injection (Shenyang Sansheng Pharmaceutical Co., Ltd., Chinese medicine prescription: S20010001), MDA Kit (Nanjing Jiancheng Bioengineering Institute, A003-1), CAT Kit (Nanjing Jiancheng Bioengineering Institute, A007-1), NO Kit (Nanjing Jiancheng Bioengineering Institute, A013-2), SOD Kit (Nanjing Jiancheng Bioengineering Institute, A001-3), Trizol (Invitrogen, 103106), reverse transcription kit (Thermo Fisher Scientific, #K1662), amplification kit (Roche, 04913914001), and IL-6 (eBioscience, BMS625) and TNF-*α* (eBioscience, BMS622) ELISA kits.

### 2.2. Animal Model Preparation and Treatment Method

A total of fifty specific pathogen-free (SPF) healthy male Sprague-Dawley (SD) rats weighing 200–220 g were provided by Shanghai Silaike Experimental Animal Co., Ltd. The rats were raised in the Experimental Animal Center of Shanghai University of Traditional Chinese Medicine in a 12/12 h light/dark cycle, with a feeding temperature of 26°C and relative humidity of 50%, and were given standard chow and water ad libitum during the experiment. After adaptive feeding for one week, the rats were divided into 5 groups randomly, with 10 rats in each group. In addition to the control group, the remaining 40 rats were given adenine 300 mg/kg/d for 10 days and administered with 250 mg/kg/d adenine by gavage for 11 days, with a total modeling period of 21 days. After modeling, the blood samples of all the animals were collected from the inner canthus. According to the creatinine values, model rats were randomly divided into 4 groups. The model group was intraperitoneally injected with 1 ml saline. The medicine including Siwu granules and EPO was administered at 10 times an adult dose by consulting references [7, 14]. The manufacturer instruction of Siwu granules recommends the dosage for adults is 5 g three times a day, and adult weight is calculated as 65 kg. The equivalent rat dosage of Siwu granules is 2.3 g/kg/d as calculated by the conversion of human dosage into the rat equivalent dose according to the respective body surface areas. The manufacturer instruction of rhEPO recommends the dosage for adults is 1000 U once a week, and the equivalent rat dosage is 1538 U/kg/w as calculated by the conversion of human dosage into the rat equivalent dose according to the respective body surface areas. The Siwu plus EPO group adopted the combination method and used the same dose of the Siwu group and EPO group. The animals received the treatment for a total of eight weeks [15]. All rats were transferred to metabolic cages in order to collect 24 h urine samples after the administration at the end of the 8th week. They were fasting and had free access to tap water for 24 h. Urine volume of rats in each metabolic cage was then recorded. At the end of the experiment, all rats were given sedation with sodium pentobarbital through intraperitoneal injection. 0.25 ml of 2% sodium pentobarbital/100 g rat weight was used to prepare and collect the blood samples and kidney tissues. Animal experiments were approved by the Experimental Animal Center of Shanghai University of Traditional Chinese Medicine.

### 2.3. Observation Index and Detection Method

The 24 h urine of rats was collected to conduct urine protein quantitative analysis, anticoagulated blood was used to perform blood routine analysis, and serum was used to measure the renal function. Analysis was completed by an automatic biochemical analyzer in the Laboratory of Shuguang Hospital Affiliated to Shanghai University of Traditional Chinese Medicine. Fresh kidney homogenate and fresh serum test oxidative stress indicators were used. The kidney tissues were fixed in 4% polyformaldehyde and embedded in paraffin for pathological staining of HE, PAS, Masson and target protein localization of TGF-*β*1 (1 : 200), *α*-SMA (1 : 200) ,EPO (1 : 100), and EPOR (1 : 100) through immunohistochemical or immunofluorescence staining. Renal tissues were stored at −80°C, and then the expressions of proteins and genes of EPO (wb 1 : 500; the primers were as follows: EPO-F: 5′ TAGCCTCACTTCACTGCTTCGG 3′ and EPO-R 5′GCGTCTGGAGGCGACATCA 3′) and EPOR (wb 1 : 500; the primers were as follows: EPOR-F 5′CCTCATCTCACTGTTGCTGACTGT 3′ and EPOR-R 5′GGTGGTGAAGAGACCCTCAAACT 3′) were detected through western blot and PCR, respectively. Inflammatory factors, IL-6 and TNF-*α*, were detected using ELISA.

### 2.4. Statistical Methods

Data are expressed as mean ± SD. The differences in the groups were analyzed by single-factor analysis of variance (one-way ANOVA). The LSD test was used to compare the differences between the two groups. The above statistical steps are completed by using statistical analysis software SPSS 18.0. The statistical significance level was set at *p* < 0.05.

## 3. Results

### 3.1. General Situation of Rats

In the modeling process, two rats died. Blood sampling from the inner canthus caused death of one rat in the control group. So, a total of three rats died before intervention. Forty-seven rats entered the stage of treatment. During the 8 weeks of treatment, one rat each from the model group and Siwu group and two rats from the EPO group died, respectively (Table 1). Compared with the model group and treatment group, the rats in the control group were sensitive, the diet was normal, the fur was dense and shiny, and the body weight was increased rapidly. However, the rats in the model group and EPO group were apathetic and inactive and had withered fur without gloss, appetite loss, and lost weight. The state of rats in Siwu and Siwu plus EPO groups was relatively active, and they had increased body weight compared with the model group and EPO group (*p* < 0.05).

### 3.2. Anemia and Renal Function in Rats with Chronic Renal Failure

**Figure 1:**
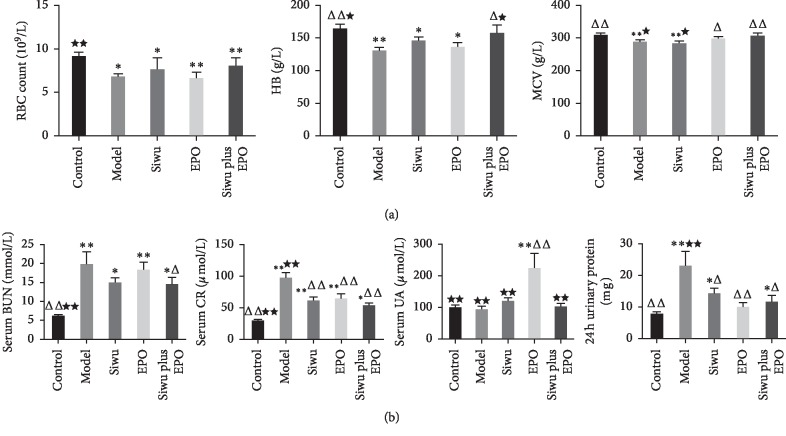
Anemia and renal function in rats with renal failure. (a) Respective and combined effects of Siwu granules and EPO on the blood routine index. (b) Therapeutic effect on renal function and 24 h urinary protein. Data are expressed as mean ± SD (*n* = 9, 9, 9, 7, 9). Compared with the control group: ^*∗*^*p* < 0.05, ^*∗∗*^*p* < 0.001; compared with the model group: ^Δ^*p* < 0.05, ^ΔΔ^*p* < 0.001; compared with the EPO group: ^*★*^*p* < 0.05, ^*★★*^*p* < 0.001.

The respective and combined effects of Siwu granules and EPO on the blood routine index are shown in Figure 1(a). Compared with the control group, the RBC of rats in other groups showed a significant decrease (*p* < 0.05). Significant differences existed between Siwu and Siwu plus EPO groups (*p* < 0.05). Siwu granules combined with EPO demonstrated a significant increase in the rat hemoglobin (HB) content (*p* < 0.05), with no differences as compared to the control group. Compared with the model group, the average hemoglobin volume (mean corpuscular volume, MCV) was significantly increased (*p* < 0.05) in the Siwu plus EPO group, and the effect was better than that of the use of Siwu granules alone (*p* < 0.05). The therapeutic effect on renal function and 24 h urinary protein quantitation is shown in Figure 1(b). Compared with the model group, the Siwu plus EPO group significantly reduced the rat blood urea nitrogen (*p* < 0.05), while the treatment group significantly reduced the serum creatinine levels (*p* < 0.001). But the effect on blood uric acid demonstrated no significant differences between the groups. The data of the EPO group were abnormal, and the standard deviation was too large. This phenomenon was either by accident or by its inherent reasons that still need to be explored. The protein excretion in 24 h urine of the model group was significantly higher than that of the control group and other medication groups (*p* < 0.05 or *p* < 0.001). After treatment, the urinary protein excretion in each group was decreased, but the EPO group showed the best effect. The reason for this result may be due to the least daily intervention in rats of the EPO group. From the perspective of anemia improvement, Siwu granules combined with EPO showed the best therapeutic effect. We supposed that Siwu granules increase the expression of endogenous EPO and reduce EPO resistance and side effects of rhEPO. Meanwhile, Siwu granules ameliorate adenine-induced kidney damage to help rhEPO increase exogenous EPO directly to promote erythropoiesis.

### 3.3. Pathological Analysis of Renal Tissue in Rats with Chronic Renal Failure

**Figure 2:**
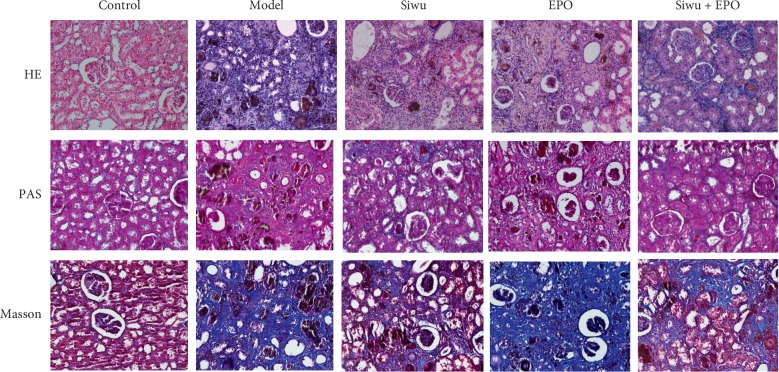
Pathological analysis of renal tissue in rats with renal failure: HE staining results of the renal tissue (magnification ×200); PAS staining results of the renal tissue (magnification ×200); Masson staining results of the renal tissue (magnification ×200).

Pathological staining of rats' kidney tissues demonstrated (Figure 2) no anomalous glomerular and renal tubular structures in the control group. Pathological manifestations of the model group are similar to those of the EPO group. The number of control glomerular cells was significantly reduced, and the structure of diseased glomeruli was unclear with a large number of brown crystals. The capsule was narrowed or disappeared with glomerular sclerosis, renal tubular necrosis and unclear structure, diffused renal interstitial collagen hyperplasia and fibrosis, diffused infiltration of macrophages, and with a large number of brown adenine crystalline structures deposition in the renal tubules and interstitium. However, the Siwu group and Siwu plus EPO group showed improvement in serious inflammatory infiltrations and severe fibrotic lesions to some extent. We supposed that the exogenous EPO could be replenished to produce red blood cells directly but has little effect on renal cell repair. In contrast, the Siwu granules either improved or retarded the renal inflammation and fibrosis to some extent and protected the renal cells.

### 3.4. Expression of EPO and EPOR in the Renal Tissues of Rats with Chronic Renal Failure

**Figure 3:**
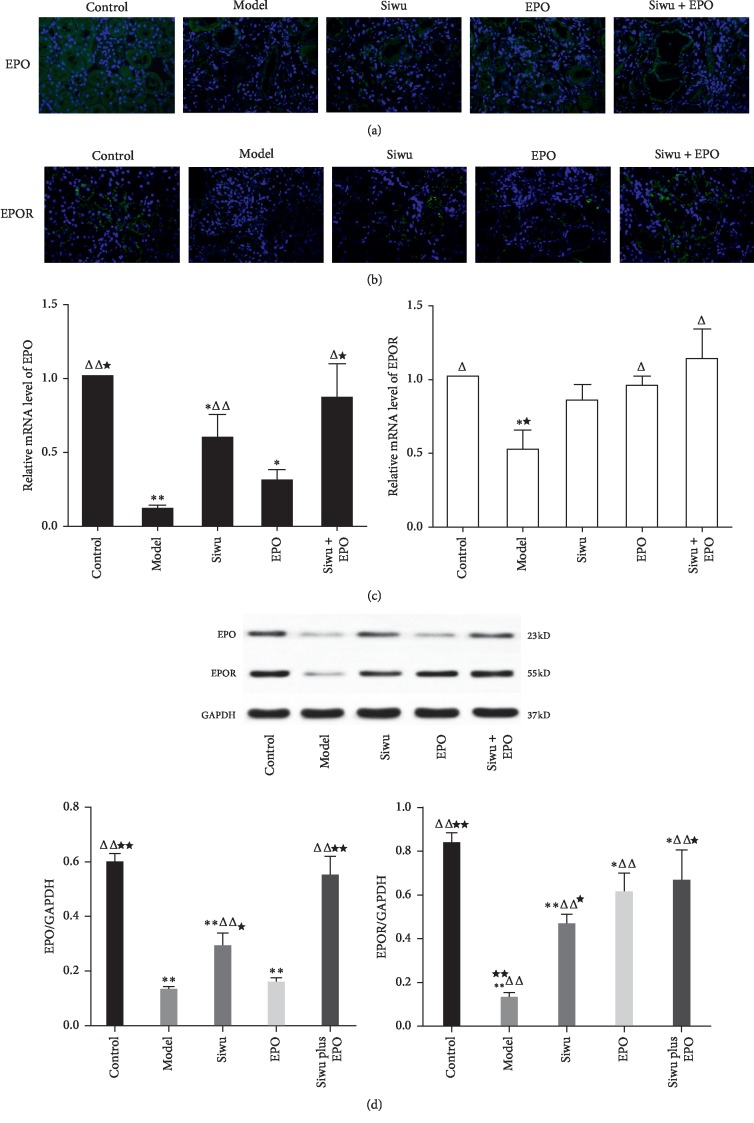
Protein and gene expression of EPO and EPOR in renal tissue of rats with renal failure: (a) immunofluorescence staining results of EPO (magnification ×400); (b) immunofluorescence staining results of EPOR (magnification ×400); (c) mRNA levels of EPO and EPOR determined through real-time PCR. Data are expressed as mean ± SD (*n* = 3). (d) Protein expression of EPO and EPOR determined through western blot. Data are expressed as mean ± SD (*n* = 3). Compared with the control group: ^*∗*^*p* < 0.05, ^*∗∗*^*p* < 0.001; compared with the model group: ^Δ^*p* < 0.05, ^ΔΔ^*p* < 0.001; compared with the EPO group: ^*★*^*p* < 0.05, ^*★★*^*p* < 0.001.

The EPO/EPOR signaling pathway is necessary for the survival, proliferation, and differentiation of erythroid progenitor cells. The effect of EPO is dependent on the concentration of EPO and the expression of EPOR. EPO originates from the mesenchymal cells adjacent to the capillaries surrounding the renal tubules. We have appraised the expression of EPO and EPOR through the supplementary immunofluorescence experiment. The study demonstrated EPO protein was expressed constitutively in the interstitial fibroblast-like cells, epithelial distal tubular cells, peritubular cells, and proximal tubular cells of normal kidneys, mostly in the outer stripe of the outer medulla and some of the cortex (Figure 3(a)). Similarly, EPOR was also expressed normally on the luminal side of proximal tubular cells in the control rat kidneys (Figure 3(b)), which is similar to that reported in Lei et al.'s research [16]. The expression levels of EPO and EPOR in the control group were the highest, and EPO and EPOR were highly expressed when treated with Siwu granules or rhEPO. RT-PCR (Figure 3(c)) and western blot (Figure 3(d)) were performed to quantitatively analyze the mRNA and protein expression levels of EPO and EPOR. Compared with the control group, rats with chronic renal failure demonstrated a significant decrease in the EPO and EPOR expression levels, while Siwu granules combined with rhEPO significantly increased EPO and EPOR levels compared with the model group, and the result was better than that when using Siwu granules or rhEPO alone.

### 3.5. Oxidative Stress in Serum and Renal Tissue of Rats with Chronic Renal Failure

**Figure 4:**
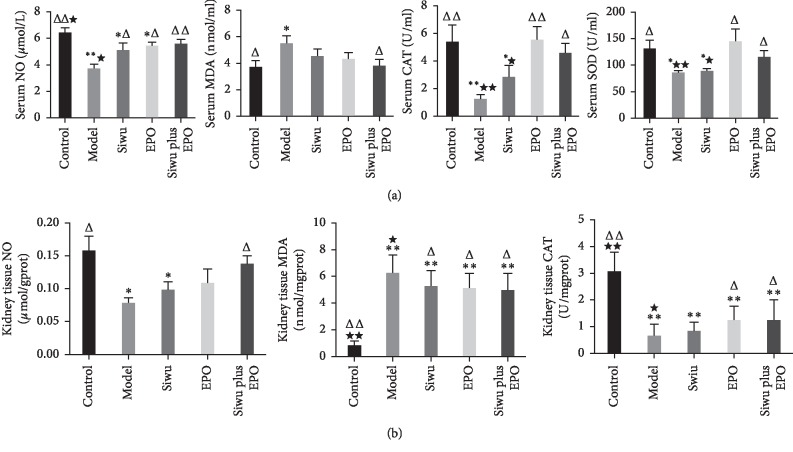
Oxidative stress in serum and renal tissue of rats with renal failure: (a) levels of nitric oxide (NO), malondialdehyde (MDA), catalase (CAT), and superoxide dismutase (SOD) in serum; (b) levels of nitric oxide (NO), malondialdehyde (MDA), and catalase (CAT) in renal tissues. Data are expressed as mean ± SD (*n* = 9, 9, 9, 7, 9). Compared with the control group: ^*∗*^*p* < 0.05, ^*∗∗*^*p* < 0.001; compared with the model group: ^Δ^*p* < 0.05, ^ΔΔ^*p* < 0.001; compared with the EPO group: ^*∗*^*p* < 0.05, ^*∗*^^*∗*^*p* < 0.001.

Anemia and oxidative stress are two common features that increase the incidence and mortality of CKD patients. CKD-related oxidative stress occurred by shortening the life of RBCs and EPO activity destruction, which in turn led to normocytic normochromic anemia. At the same time, renal anemia itself produced more free radicals because of increased aerobic metabolism and weakened the ability of the antioxidant system to remove the superoxides. Moreover, the conventional treatment of iron supplementation also strengthened the oxidative stress in the kidney [17]. Therefore, anemia and oxidative stress are involved in the reciprocal causation and commonly aggravate renal damage in CKD. In this study, we examined the levels of nitric oxide (NO), malondialdehyde (MDA), catalase (CAT), and superoxide dismutase (SOD) in serum and renal tissues of rats with adenine-induced chronic renal failure. In the serum oxidative stress indices (Figure 4(a)), each treatment group improved the antioxidant and oxidative balance, especially with the combination of Siwu granules and rhEPO. This combination significantly increased the serum NO (*p* < 0.001), CAT (*p* < 0.05), and SOD (*p* < 0.05) levels and reduced MDA (*p* < 0.05) levels. The effect of Siwu granules on suppressing the oxidative stress was better than that of EPO. Under oxidative stress in renal tissues (Figure 4(b)), we found that compared with the model group, only the Siwu plus EPO group demonstrated increased NO levels and showed significant differences in the kidney (*p* < 0.05). MDA was increased (*p* < 0.001) and CAT was decreased (*p* < 0.001) in renal tissue of adenine-induced CKD model rats. Each treatment group had elevated renal CAT (*p* < 0.05) and depressed renal MDA (*p* < 0.05) levels. Qiang-Ming Li et al. also found oxidative stress damage in the adenine-induced renal failure rat model, and the alteration of SOD, GSH, and MDA levels could be remarkably reversed by Chinese chive polysaccharides, a common gradient in the traditional Chinese medicine in a dose-dependent manner [18]. Our results suggested that the enhancement of antioxidant ability might be one of the mechanisms required for Siwu and rhEPO to protect renal function.

### 3.6. Expression of IL-6 and TNF-*α* in the Kidney of Rats with Chronic Renal Failure

**Figure 5:**
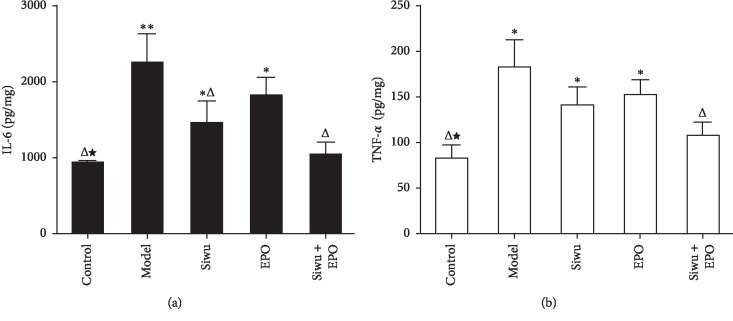
Expression of (a) IL-6 and (b) TNF-*α* in kidney tissues of rats with renal failure through ELISA. The data are expressed as mean ± SD (*n* = 6). Compared with the control group: ^*∗*^*p* < 0.05, ^*∗∗*^*p* < 0.001; compared with the model group: ^Δ^*p* < 0.05, ^ΔΔ^*p* < 0.001; compared with the EPO group: ^*★*^*p* < 0.05, ^*★★*^*p* < 0.001.

In CKD, inflammation and oxidative stress are two aspects that accelerate the progression of the disease. In this study, we examined IL-6 (Figure 5(a)) and TNF-*α* (Figure 5(b)) expression levels in the rat kidney, and the results revealed significantly higher expression levels in the model group than the control group and the treatment groups. rhEPO and Siwu granules lowered the levels of inflammatory factors but showed no statistically significant differences between the groups.

### 3.7. Immunohistochemical Analysis of Renal Fibrosis Indices TGF-*β*1 and *α*-SMA in Rats with Chronic Renal Failure

**Figure 6:**
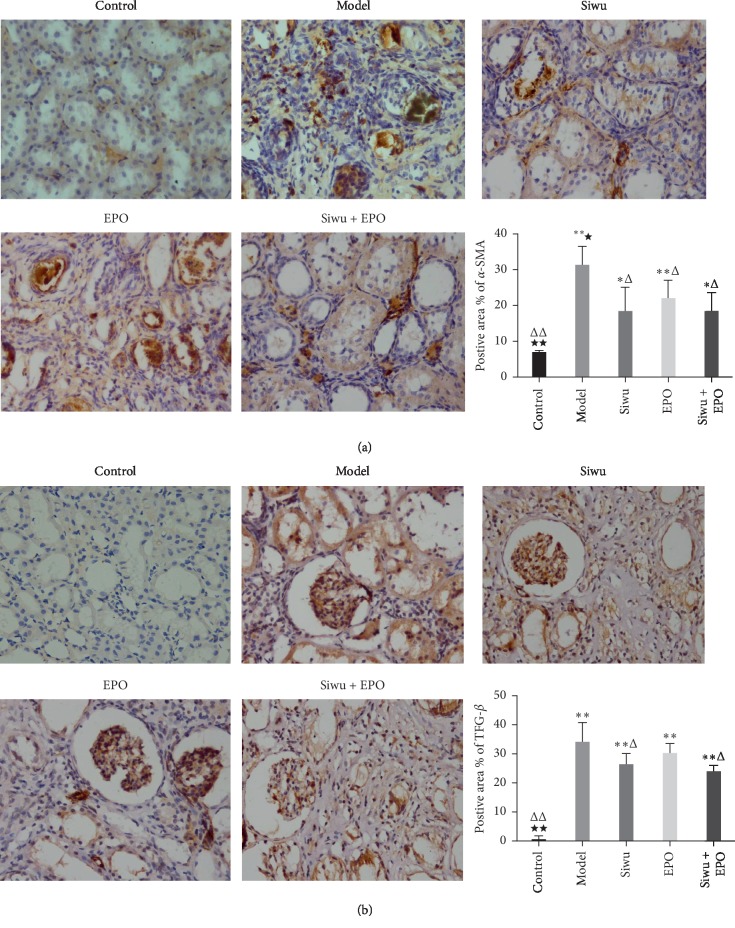
Immunohistochemical analysis of renal fibrosis indices *α*-SMA and TGF-*β*1 in rats with renal failure: (a) immunohistochemical staining results of *α*-SMA (magnification ×400); (b) immunohistochemical staining results of TGF-*β*1 (magnification ×400). The data are expressed as mean ± SD (*n* = 3). Compared with the control group: ^*∗*^*p* < 0.05, ^*∗∗*^*p* < 0.001; compared with the model group: ^Δ^*p* < 0.05, ^ΔΔ^*p* < 0.001; compared with the EPO group: ^*★*^*p* < 0.05, ^*★★*^*p* < 0.001.

Renal fibrosis is the key reason for the development of CKD to ESRD, which involves excessive deposition of the extracellular matrix (ECM), leading to the destruction of the renal structure and progressive declination of renal function. Oxidative stress and inflammation play an important role in the process of renal fibrosis. Inflammatory cell infiltration and aggregation, inflammatory cytokines, and promoting fiber factors stimulate the formation of myofibroblasts, a necessary step that is involved in the development of renal fibrosis. Persistent oxidative stress and inflammation can stimulate various cytokines and activate multiple signaling pathways, which play an important role in the occurrence and development of renal fibrosis. A study [19] by Asada et al. described the relationship between renal anemia and renal fibrosis. In the CKD process, fibroblasts that generate EPO were produced by epithelial mesenchymal transition (EMT) from damaged renal tubular epithelial cells. These further transdifferentiate into myofibroblasts, which directly promote the formation of fibrosis, and this process is accompanied by decreased EPO production. PAS and Masson staining of kidneys showed a strong collagen deposition and diffused fibrosis in the kidney of rats with adenine. In order to analyze the effects of Siwu granules and rhEPO on renal fibrosis, we performed the fibrosis classic index of TGF-*β*1 (Figure 6(a)) and mesenchymal marker protein *α*-SMA expression (Figure 6(b)) through immunohistochemical analysis. Positive staining was observed and showed higher expression levels of TGF-*β*1 and *α*-SMA in the glomerulus and tubulointerstitium of chronic renal failure rats, including glomerular epithelial cells, vascular endothelial cells, mesangial cells, renal tubular epithelial cells, and renal interstitial fibroblasts. In contrast, no expression was observed in the control group, which can be improved after treatment. The immunohistochemistry positive area statistics showed that the expression of *α*-SMA and TGF-*β*1 in the model group was significantly higher than that of the control group. Compared with the EPO group, there was no difference between the treatment groups, and each treatment group could reduce the expression of *α*-SMA, but TGF-*β*1 expression in the EPO group and model group had no statistical significance. The result is consistent with that of Lea Pedersen et al.'s experiment, who observed that EPO treatment in the anemic model of chronic kidney disease normalises hemoglobin levels but has no effect on kidney fibrosis or function, even if the treatment with EPO is prolonged [20]. Furthermore, TGF-*β*1 upregulated the collagen I protein expression [21]. Deposition of TGF-*β*1 and *α*-SMA may lead to renal fibrosis through epithelial mesenchymal transdifferentiation and endothelial mesenchymal transdifferentiation.

## 4. Discussion

Adenine is a common drug used to induce a chronic renal failure model. This is because 2,8-dihydroxyadenine that is produced by adenine metabolism is extremely difficult to dissolve in the urine to be excreted and hence is blocked in the renal tubule. This in turn causes tubulointerstitial nephritis, leading to chronic renal failure. Akchurin et al. [15] successfully induced chronic renal failure model with high adenine diet. Serum iron was decreased and IL-6 and TNF-*α* levels were elevated in the adenine diet mice. Nemmar et al. [22] induced the rat model of chronic renal failure with adenine and demonstrated significantly increased levels of TNF-*α*, lipid peroxides, and ROS. Histopathology showed more collagen deposition, more neutrophil infiltration, tubular dilation, and glomerular damage. In this study, we established chronic renal failure in the rat model by inducing adenine. Compared to nephrectomy, there was less injury for rats and no inflammatory response occurred in the postoperative wound healing of the current model. After modeling, except in the control group, one rat died accidentally due to blood sampling and a total of two rats were killed. The results showed that the modeling method was feasible. In the process of drug treatment for 8 weeks, 2 rats died in the EPO group, and the mortality rate is significantly higher than that of the Siwu group and Siwu plus EPO group.

Our research showed better therapeutic effect of Siwu granules and rhEPO on anemia and renal function. Ribeiro et al. [23] observed that renal function and anemia in 5/6 nephrectomized rat models demonstrated kidney damage, low oxygen, renal fibrosis, and inflammation with different doses of rhEPO. These results showed great benefits on kidney inflammation with high doses of rhEPO and did not affect the renal function. These results indicate that Chinese medicine Siwu granules combined with rhEPO showed great benefits on kidney damage and produced the feeble state of rats. This strengthened the rat and prolonged the survival period or postponed the dialysis time in patients with chronic renal failure.

EPO is mainly produced in the kidney and secreted into the blood circulation. This in turn targets bone marrow erythroid progenitor cells and then stimulates the formation of RBCs. Hemoglobin in RBCs is responsible for oxygen transportation, and so, the main function of EPO is through the production of RBCs that regulate the transportation of oxygen, especially under hypoxic conditions for EPO gene transcription. Hypoxia-inducible factor (HIF) is a major transcription factor that regulates EPO production. In addition, hepatic nuclear factor-4 (HNF-4), retinoic acid receptor alpha (RXR-alpha), SMAD3, and Sp1 also regulate the transcription factors [24]. The work performed by Rivkin and his colleagues has uncovered the mechanism that inflammation increases blood levels of MIR122, by which the expression of EPO in the kidneys is reduced and anemia is caused. Strategies to block MIR122 in patients with inflammation could reduce the development or progression of anemia [25].

The activity of EPO is determined by its wide division in the cell surface receptor, i.e., EPOR. EPOR expression was observed not only in the erythroid progenitor cells but also in a variety of other nonhematopoietic tissues [26]. The combination of EPO and EPOR in nonhematopoietic tissues plays a cytoprotective role, including antioxidative stress, inhibition of apoptosis, and mobilization of endothelial progenitor cells to promote angiogenesis repair. Dang et al. [27] confirmed the expression of EPOR in renal tubular epithelial cells by immunofluorescence and immunohistochemistry. EPO can inhibit the oxidative stress and apoptosis of renal tubular cells induced by high glucose, which is in turn regulated by EPOR. Hertzberg-Bigelman et al. [28] established the CKD model through nephrectomy. The animal model demonstrated a decreased creatinine clearance rate, increased blood urea nitrogen levels, and anemic symptoms of chronic renal failure. Renal pathology also showed strong renal fibrosis after 11 weeks. Comparison of these changes demonstrated that although the level of EPO in the heart was similar, EPOR and STAT5 downstream molecules were reduced significantly, indicating CKD as the cause of molecular changes at the EPO/EPOR axis. EPO/EPOR signaling plays a protective role in multiple organs and tissues such us adipose tissue, pancreas, heart, skeletal muscle,and nervous system and pathological condition such as obesity, diabetes, and cancer [29]. Studies have confirmed that EPOR is located in human, rat, and mouse glomerular, mesangial, and tubular epithelial cells in the kidney. EPO/EPOR not only stimulates the generation of RBCs but also demonstrates the functions of anti-inflammation and antiapoptosis, inhibiting oxidative stress and promoting the regeneration of tubular epithelial cells and vascular endothelial cells. Souza et al. [30] confirmed that EPO can inhibit the oxidative stress and inflammatory response and protect the renal function by regulating the expression of NF-kB, eNOS, and EPOR in acute kidney injury. Wang et al. [31] found that rhEPO could inhibit the mRNA expression of nicotinamide adenine dinucleotide phosphate (NADPH) oxidase and NADPH oxidase-dependent superoxide production in streptozotocin- (STZ-) induced diabetic rats and also has multiple effects of antioxidation and anti-inflammation. A cross-sectional study conducted by Dimitrijevic et al. [32] reported that the long-term use of EPO can alleviate uremia/renal anemia-related oxidative stress damage. Patients with ESRD receiving long-term peritoneal dialysis were treated with EPO and demonstrated significant reduction in the production of ROS, thereby reducing the number of white blood cells that play an anti-inflammatory role [33]. Li et al. [34] also found that oxidative stress is the main reason for the decrease of EPO expression in aging of rats. In addition to the inhibition of oxidative stress and inflammation, EPO can also inhibit fibrosis of renal tubular epithelial cells by inhibiting EMT, where EPO downregulates the expression of TGF-*β*, *α*-SMA, fibronectin, and connective tissue growth factor [35]. However, many original research studies showed that soluble EPOR levels may contribute to erythropoietin resistance in ESRD and that soluble EPOR production may be mediated by proinflammatory cytokines [36].

Therefore, we supposed that TCM can increase the expression of endogenous EPO on renal anemia and has a better therapeutic effect, or reduce EPO resistance and side effects of drugs with additional exogenous EPO. For example, Mao et al. [37] reported that the Huangkui capsule can inhibit the oxidative stress and activity of p38MAPK signaling pathway in renal tissues of diabetic nephropathy and improve renal fibrosis. Many studies have also demonstrated the anti-inflammatory and antifibrotic effects of Siwu decoction in the treatment of various diseases. Tang et al. [38] studied the contribution of aromatic acids to the antioxidant activity of their related prescriptions, which proved that the radical-scavenging activity of Siwu decoction was the strongest. The main components of Siwu decoction included paeoniflorin and senkyunolide I, which not only showed antiplatelet and anticoagulation activities but also indirectly contributed to the total bioactivity reflection of Siwu decoction. Especially the latter has drawn attention for the research of complicated bioactive constituents of TCM or its formula [39]. Wang et al. [14] demonstrated that Siwu decoction exhibited antihyperuricemia and anti-inflammatory effects by inhibiting hepatic XOD activity. This consequently regulates the renal organic ion transporter expression, suppresses renal NLRP3 inflammasome activation, and provides the evidence for its use in the treatment of hyperuricemia and its associated kidney inflammation.

Chronic renal failure patients are more commonly observed with the microinflammatory state, metabolic disorders, oxidative stress, and glycation end product augmentation. These in turn increase the inflammatory proteins and reduce the renal clearance, with the further increase of the inflammatory cytokines in the kidney. Almroth et al. [40] demonstrated that the peritoneal dialysis patients had higher levels of inflammatory cytokines, IL-6 and TNF-*α*, and fibroblast growth factor 23 (FGF23) and high sensitivity CRP compared with the healthy crowd, which is a direct proof of a high inflammatory state in patients with chronic renal failure. In the early stage of kidney disease, iron status is associated with renal inflammation [41]. The inflammatory cytokines can increase erythropoietin resistance, inhibit erythropoiesis, accelerate the destruction of RBC/hemoglobin, suppress the antiapoptotic activity of EPO, increase iron consumption, decrease serum iron and transferrin concentrations, and aggravate renal anemia due to iron deficiency. Researchers [25] found that LPS-induced renal inflammation in mice can increase the miR-122 levels in the blood and decrease the EPO expression in the kidney, which may be one of the mechanisms of inflammation that induce anemia. Our experimental results also demonstrate the therapeutic effect through inhibiting inflammation on renal anemia.

## 5. Conclusions

In short, this study used the combination of Chinese and Western medicine and by experimentation proved that Siwu granules combined with EPO significantly upregulated the EPO/EPOR signal, so that Siwu granules and EPO together can play a more powerful function as antioxidative damage, anti-inflammation, and antirenal fibrosis, and synergistically ameliorated anemia in adenine-induced chronic renal failure rats.

## Figures and Tables

**Table 1:** Mortality and body weight of rats in each group.

Group	*N*	Death number	Mortality (%)	Body weight (g)
Control	9	0	0	444.00 ± 11.81^Δ^
Model	10	1	10	380.67 ± 15.89^*∗*^
Siwu	10	1	10	390.78 ± 17.99^*∗*^
EPO	9	2	22	380.14 ± 9.84^*∗*^
Siwu plus EPO	9	0	0	392.00 ± 17.70^*∗*^

Compared with the control group: ^*∗*^*p* < 0.05; compared with the model group: ^Δ^*p* < 0.05.

## Data Availability

The data used to support the findings of this study are available from the corresponding author upon request.
